# Going the Distance: Ethical Issues Arising When Patients Seek Cancer Care From International Settings

**DOI:** 10.1200/JGO.17.00001

**Published:** 2017-06-09

**Authors:** Daniel J. Benedetti, Mehra Golshan, Jennifer C. Kesselheim

**Affiliations:** **Daniel J. Benedetti** and **Jennifer C. Kesselheim**, Dana Farber/Boston Children’s Cancer and Blood Disorders Center; and **Mehra Golshan**, Dana Farber Cancer Institute and Brigham and Women’s Hospital, Boston, MA.

Medical tourism is the well-described phenomenon in which patients travel to less developed countries for medical care. Far less is written about the growing practice of international patients traveling to developed countries for second opinions, research, or specialized care that is unavailable in their home countries. Because cancer care represents a major component of this phenomenon, we became motivated to examine the unique ethical challenges of this practice. In this commentary, we identify and discuss ethical questions arising in the care of patients from international settings and make suggestions for future work to be done in this evolving area of cancer medicine.

## Arrival-Related Issues

Vignette: CX is a 30-year-old man from China referred for treatment of acute leukemia. The accepting oncologist, who had no direct contact with the patient or referring physician, remains uncertain about the patient’s treatment history. On arrival, records and history provided by the patient reveal that he has already undergone two stem cell transplantations. He insists that he was told there would be curative treatment options available.

The international patient referral process requires many intermediaries and typically occurs without direct communication among the patient, referring physician, and accepting provider. Flaws in the intake system may yield challenges to general ethical principles, including (1) the commitment to the best interests of the patient, (2) respect for patient autonomy, (3) the recognition and mitigation of conflicts of interest, and (4) safeguarding fairness toward the total population of patients with cancer.

### Impediments to optimal care.

Providers accepting patients from international settings may feel forced to provide care that does not meet their usual standards, causing moral distress.^1^ First, the accepting physician initially functions without complete information because transmitted records may be untranslated or incomplete, and some data may be unavailable until the patient transports them. Second, the inevitable delay between patient acceptance and arrival can be detrimental and may change the disease stage, treatment options, and prognosis. Accepting providers may make poor decisions when they lack accurate data.

Patients transferring from international settings may receive care that is less efficient. With incomplete records and no contact with the referring provider, the reviewing oncologist must either (1) request additional documentation, further delaying arrival; or (2) accept the patient and review the available data when the patient brings it. Both options are inefficient, and the oncologist may feel distressed about compromising this core dimension of quality of care.^2^

### Informed consent.

Patient autonomy holds a revered place in Western medical ethics and clinical decision making. Informed consent, which protects patient autonomy, comprises five key components: (1) capacity, (2) disclosure, (3) understanding, (4) voluntariness, and (5) decision.^3^ Adequate disclosure requires the provision of information about the diagnosis, the reason for a proposed intervention, anticipated benefits, potential risks, and acceptable alternatives. Inadequate communication between the accepting oncologist and the patient before patient arrival can undermine informed consent by hindering both disclosure and understanding. When informed consent quality is compromised, patients may receive care incongruent with their genuine preferences.

### Conflicts of interest.

Decisions about whether to accept international patients are vulnerable to conflicts of interest. International patients who are wealthy or come from wealthy nations constitute a significant financial opportunity for institutions, which may result in either implicit or explicit pressure on the physicians reviewing patients for acceptance. Furthermore, embassy medical attachés may send a patient’s file to multiple institutions, forcing the reviewer to make a quick decision before a competing institution accepts the patient. These pressures may unduly influence an institution’s review process or even an individual reviewer’s decision.

### Justice.

The principle of justice means that equals should be treated equally, and distributive justice suggests that scarce resources ought to be distributed according to principles of fairness.^4,5^ The care of international patients tends to be resource intensive. Interpreters or cultural navigators are needed more frequently than for local patients. Care coordination with embassies is more complex than with traditional insurance companies. Prescriptions may require unfamiliar prior authorization processes, and home-care needs or ancillary services such as physical therapy may take longer to arrange. For this patient population, hospitalizations may be longer than medically indicated, and care proves more time consuming. Given that hospital beds and clinician time represent finite resources, international patients can raise questions of distributive justice.

### Recommendations.

- Institutions should improve and standardize the patient intake process, involving key stakeholders to develop an efficient yet thorough process to facilitate (1) timely transmission of accurate, translated, and comprehensive information; (2) direct communication with the referring provider; and (3) a conversation with the patient.
- Whether conflict of interest concerns are legitimate is unknown; future studies could investigate whether they exist and, if so, how they impact patient acceptance.
- Institutions should engage in reflective discussion about how obligations to the local community integrate with the care of international patients. Data on resource utilization, hospital length-of-stay, and provider and staff time could reveal whether and when international patient volume might compromise care for other patients and whether volume limits should be considered.
- Future studies could examine whether profit from the care of international patients truly allows institutions to provide services to all patients that would be otherwise unavailable or unaffordable.

## On Arrival

Vignette: SA is a 22-year-old woman from the Kingdom of Saudi Arabia referred for treatment of osteosarcoma of the distal femur. Doctors in Saudi Arabia recommended chemotherapy with an amputation as local control. Seeking limb-sparing surgery in the United States, the family delayed initiation of chemotherapy to transfer care. The delay caused tumor growth that made limb salvage impossible. Before you can share this news, her father takes you aside and states his firm preference that his daughter not be informed of the details of her condition.

After a patient arrives for cancer care, ethical challenges can arise from misaligned expectations regarding the treatment plan and its goals, and from conflicts related to differences in culture.

### The role of culture.

In Western medicine, where patient autonomy is of utmost importance, adequate disclosure requires us to provide patients with details about their prognosis and anticipated adverse effects of their treatment. Yet, in some cultures, communication flows through a family member, who may keep details from the patient. Western clinicians, trained to embrace honest disclosure, may experience moral distress as their ethical principles come into conflict with cultural norms of the patient and his or her family.

### Treatment goals.

Patients arriving from international settings may hold misunderstandings about their tumor, prognosis, and recommended treatment. Patients may arrive with more advanced disease than originally thought, or the accepting team may interpret patient data differently, causing recommendations to deviate from what the patient expected. Patients may also have unrealistic expectations about the care available in Western countries, believing that newer technology or better medicines exist and will improve outcomes when, in reality, treatment recommendations and prognostic data may mirror those from the home country. The patient endures enormous inconvenience and investment of time and resources to travel to his or her destination country, burdens that create pressure to begin treatment expeditiously on arrival. Without first addressing misunderstandings and reaching a shared vision of treatment goals on the basis of a realistic picture of the patient’s diagnosis and prognosis, patients may experience frustration or dissatisfaction, especially if their goals are not met.

### Impediments to optimal care.

Having the embassy in lieu of an insurance company can present new problems delivering optimal care. Physicians are accustomed to approval processes for domestic health insurers, but embassies operate differently and sometimes without the transparency expected in Western settings. Many providers report delays in obtaining approval for services and express frustration with the lengthy approval and appeal processes that can delay needed care, leading to patient harm.

### Recommendations.

- Improved communication before patient arrival may alleviate some mismatched expectations. Institutions should develop resources explaining key differences in health care between countries, which should be incorporated into the patient’s decision of whether to pursue care abroad, in keeping with the principle of informed consent.
- Accepting oncologists should engage in thorough and honest communication about the patient’s situation, leaving open the possibility that treatment at the new institution may not be the best option.
- Oncologists may require support in understanding and navigating the cultural differences between countries. Cultural navigators may be helpful members of the care team, and interdisciplinary team meetings, including international case coordinators, may allow for improved communication and streamlined care. Clinicians should be supported and encouraged to improve their cultural competency, because the benefits will extend to all patients, not just international ones.
- Institutions should work with embassies to streamline communication and provide transparency about coverage, because oncologists may erroneously assume that everything typically included in Western cancer care is approved when a patient’s care is embassy supported.

## Departure-Related Issues

Vignette: MA is a 45-year-old man from the United Arab Emirates referred for treatment of lymphoma. He received chemotherapy in a US cancer center but experienced multiple complications, and on completion of therapy, he still requires frequent monitoring and active management. Despite this, his embassy insists that he return home immediately and informs him that he will have to pay out of pocket for all subsequent care in the United States.

International patients typically return home after completing planned therapy but may also depart during treatment when an embassy withdraws financial guarantee or when no further curative options are available. Sending patients back to foreign countries presents unique challenges.

### Best interests.

Although it is important that departing patients reconnect with a local oncologist as soon as possible, ensuring this may be difficult. For the patient receiving active chemotherapy or managing toxicities, such as patient MA, the lack of advance warning about coverage termination may force the patient to travel at a potentially unsafe time. Transition back to the home country may be fraught with complications if communication with a local oncologist is inadequate or unavailable. The best option is often to give the patient a letter summarizing the care received and follow-up recommendations, yet this places the onus on the patient to ensure that follow-up occurs. The treating institution and physician have a moral duty to the patient, which is compromised by allowing an unsafe transfer of care.

### Justice.

Medical visas are a scarce resource in international health care: countries have a finite number for their citizens. Embassies may limit the length of care they initially approve, even if the planned treatment course is longer, requiring reapproval to extend coverage. On completion of therapy, many patients returning home wish to return to the Western cancer center for follow-up and ask the oncologist for a letter with this as a recommendation. As the number of patients pursuing international treatment increases, rationing decisions must be made with regard to patient acceptance and treatment approval.

Embassies may instruct patients without curative options to return home; however, palliative and hospice care abroad may not exist or approximate that which exists in Western countries. The demand for medical visas directly pits the interests of the patient with end-of-life care needs against those of the patient who may benefit from curative treatment. Utilitarian arguments^5,6^ can be made regarding who should receive the visa; however, this approach fails to account for moral distress caused by forcing the patient to return home and receive less-than-ideal end-of-life care.

### Recommendations.

- Efforts to standardize intake communication should also facilitate efficient and safe transfers of care back to patients’ home countries.
- Institutions should negotiate assurances from foreign embassies that services will be covered for the duration needed to guarantee patients’ safe return home. Such a strategy is essential to ensure that patients’ safety will not be compromised by premature or poorly timed relocation back to their home country.

In conclusion, the increase in patients traveling internationally for cancer care poses new challenges for oncologists. Although the same bioethical principles apply to the care of domestic citizens and international patients, we have highlighted a handful of unique ethical dilemmas that have emerged as this practice expands and have offered some suggestions for future research and resource allocation. Given the financial incentives to recruit international patients, these issues and others will require more attention from researchers and institutional leaders who coordinate international patient care.
